# Comparing the Effects of Integrative Neuromuscular Training and Traditional Physical Fitness Training on Physical Performance Outcomes in Young Athletes: A Systematic Review and Meta-Analysis

**DOI:** 10.1186/s40798-025-00811-2

**Published:** 2025-02-08

**Authors:** Ke-wen Wan, Zi-han Dai, Po-san Wong, Robin Sze-tak Ho, Bjorn T. Tam

**Affiliations:** 1 Academy of Wellness and Human Development, Faculty of Arts and Social Sciences, Hong Kong Baptist University, Kowloon Tong, Hong Kong, China; 2 Dr. Stephen Hui Research Centre for Physical Recreation and Wellness, Hong Kong Baptist University, Kowloon Tong, Hong Kong, China; 3 Department of Sports Science and Physical Education, The Chinese University of Hong Kong, Hong Kong, China

**Keywords:** Integrative neuromuscular training, Physical fitness training, Young athletes, Physical performance

## Abstract

**Background:**

Enhancing youth's physical fitness levels during childhood and adolescence through prior training programs can significantly optimize their athletic performance. The integrative neuromuscular training (INT) model is designed to improve neuromuscular function and prevent athletic injuries, and is superior to the traditional physical training method. This systematic review and meta-analysis aims to compare the effects of INT versus traditional physical fitness training (PT) on physical performance-related outcomes in young athletes.

**Methods:**

Six online databases (PubMed, MEDLINE, Embase, SPORTDiscus, Web of Science, PsycINFO) searched from inception through 15 January 2024. Meta-analysis was performed when data were available from two or more trials. Physical performance-related outcomes were analyzed using standardized mean differences (SMDs) and mean differences (MDs) with 95% confidence intervals (CIs). The certainty of evidence and quality of the included studies were rated using the GRADE scale and the revised Cochrane risk-of-bias tool respectively.

**Results:**

Seventeen randomized controlled trials with 649 young athletes were included in the systematic review. Of those, 1 study showed a low risk of bias, 1 showed a high risk, and 15 showed some concerns. Compared to the PT group, INT significantly improved dynamic balance (MD = 7.29%, 95%CI 3.31 to 11.28, *p* < 0.001, I^2^ = 64.7%), static balance (SMD = −0.54, 95%CI −0.84 to −0.24, *p* < 0.001, I^2^ = 0.0%), jumping performance (SMD = 0.53, 95%CI 0.32 to 0.73, *p* < 0.001, I^2^ = 0.0%), sprinting capacity (SMD = −0.76, 95%CI −1.13 to −0.39, *p* < 0.001, I^2^ = 57.6%) and maximal strength (SMD = 1.01, 95%CI 0.35 to 1.67, *p* = 0.002, I^2^ = 81.9%%). No significant between-group differences were identified for agility and flexibility.

**Conclusion:**

Our findings suggest that INT has the potential to be an effective training method to improve physical performance in young athletes. Moreover, further research is needed to determine the effects and associated doses for young athletes of different age groups.

**Supplementary Information:**

The online version contains supplementary material available at 10.1186/s40798-025-00811-2.

## Key Points

- Integrated neuromuscular training (INT) significantly improves balance, lower-body explosive power, and maximal strength in young athletes compared to traditional physical fitness training.
- INT interventions show significant enhancements in agility and flexibility after sensitivity analysis, indicating their potential for comprehensive physical performance improvements.
- Subgroup analysis suggests that longer interventions (over 12 weeks) and higher training frequencies (triweekly) show a trend towards greater improvements in young athletes' physical performance, despite not reaching statistical significance.

**Supplementary Information:**

The online version contains supplementary material available at 10.1186/s40798-025-00811-2.

## Background

Enhancing youth's physical fitness levels during childhood and adolescence through prior youth-based training programs has been shown to significantly optimize their athletic performance in adulthood [1–3]. This is particularly important in preventing early-onset diseases, as improved physical fitness during childhood has substantial long-term health benefits in adulthood [4, 5]. Furthermore, studies indicate a critical "window of opportunity" for accelerated adaptation to motor coordination between the ages of nine and twelve, during which young athletes exhibit heightened responsiveness to training-induced adaptations. [6]. However, it is important to note that young athletes who commence athletic training with limited strength and conditioning experience may have compromised neuromuscular control, which can negatively impact fundamental movement patterns and biomechanical alterations [7, 8]. This, in turn, can lead to poor physical performance and an increased risk of musculoskeletal injuries. Therefore, in light of this information, implementing an effective training strategy is crucial for enhancing the development and performance of youth.

Physical performance is a complex construct influenced by multiple factors, including fitness components such as strength, speed, endurance, flexibility, and skill [9, 10]. The integrative neuromuscular training (INT) model is specifically designed to enhance these components by improving neuromuscular function, muscular strength, and capacity for sport-specific activities [11, 12]. Unlike neuromuscular training, which solely targets improving neuromuscular control and coordination, INT stands out as a more comprehensive training modality. It encompasses two distinct segments, namely basic motor skills and sport-specific skills, offering a holistic approach to enhancing athletic performance [13]. The basic motor skills segment of the INT model focuses on cultivating coordination, strength, augmentation, agility, dynamic stability, speed, and fatigue resistance, while the second segment emphasizes the acquisition and refinement of sport-specific skills, enabling performance optimization within the context of individual sports [13]. The primary objective of INT is to enhance the quality of athletes' functional movements by addressing neuromuscular control deficits and preventing athletic injuries. Through the effective improvement of specialized movement patterns and the enhancement of movement efficiency, INT ultimately enables athletes to achieve optimal athletic performance [12, 14, 15].

Previous studies have demonstrated that INT compared to traditional neuromuscular training of a single trunk and muscle group has better overall results in enhancing proprioception and spatial localization in specialized sports [16]. Specifically, compared to traditional physical fitness training (PT), INT places greater emphasis on the development of neuromuscular innervation, and the strengthening of proprioception, which are crucial for preventing injuries and optimal physical performance. However, despite the promising findings, there are remaining inconsistent results from randomized controlled trials (RCTs) with respect to physical performance. While several RCTs have demonstrated that INT can lead to significant improvements in adolescent motor skills and basic physical fitness when compared with PT [12, 14, 15], it is important to recognize the presence of conflicting results. In certain RCTs, no improvements or potentially adverse effects on particular physical performance outcomes, such as agility and flexibility, have been reported [16, 17]. These divergent findings underscore the necessity for a thorough evaluation to gain a more comprehensive understanding of the overall effects of INT versus PT on various physical performance metrics.

To our understanding, there is currently no systematic review with meta-analysis that specifically contrasts INT and PT interventions among young athletes. Although a previous systematic review has examined the effects of INT on injury prevention and performance [10], it lacked detailed descriptions of intervention protocols and quantitative data analysis methods. Additionally, the review did not examine the effects of INT on different physical fitness outcomes. The aim of the current systematic review and meta-analysis was to consolidate and analyze the existing evidence in order to offer a more comprehensive assessment of the effects of INT and PT on physical performance outcomes in young athletes.

## Methods

### Study Design

This systematic review with meta-analysis was performed following the Preferred Reporting Items for Systematic Reviews and Meta-Analyses (PRISMA) [17, 18] recommendations and was registered on the International Prospective Register of Systematic Reviews (PROSPERO) (identification code: CRD42023465948).

### Data Sources and Search Strategy

A comprehensive literature search was conducted on May 6, 2024, using six electronic databases: MEDLINE, PubMed, Embase, SPORTDiscus, Web of Science, and PsycINFO. The search was limited to full-text articles published in English and involving human subjects. The search strategy is provided in Table S1. Endnote (Clarivate Analytics) was used to import all search results, and any duplicates were removed. Two researchers (KW, ZD) independently performed title, abstract screening, and full-text screening of each article, and no automated or semi-automated approaches, including machine learning-based methods, were utilized for record screening. According to the PRISMA guidelines, duplicates were removed using the EndNote software. A third independent reviewer (RH) was consulted to settle any discrepancies in the results. Furthermore, we manually searched the reference lists of articles in the final analysis. Additionally, the reference lists of relevant reviews, systematic reviews, and meta-analyses were screened, along with the reference lists of the included articles, to ensure comprehensive coverage.

### Eligibility Criteria

Two authors (KW and ZD) conducted independent screening of all papers to determine eligibility, guided by the following pre-defined inclusion and exclusion criteria:

Inclusion Criteria:

- Participants: Young, healthy athletes aged 8 to 21 with various sports backgrounds [19].
- Interventions: Studies utilizing INT as the experimental intervention, incorporating fundamental movement skills (e.g., coordination, strength, plyometric, agility, dynamic stability, speed, and anti-fatigue training) and sport-specific skills [11].
- Comparators: Studies comparing INT with PT, defined as conventional exercise programs aimed at enhancing overall physical fitness and sports skills.
- Outcomes: Studies reporting on physical performance-related outcomes, such as strength, power, agility, balance, and flexibility.
- Study design: Randomized controlled trials or randomized crossover trials.

Exclusion Criteria:

- Studies involving participants with chronic health conditions or injuries.
- Non-randomized studies or observational research.
- Interventions not primarily focusing on neuromuscular or physical fitness training.
- Studies not providing sufficient data for analysis.

### Data Extraction

The data extraction procedures followed the guidelines outlined in the Cochrane Collaboration Handbook [20]. The data extraction was completed independently by two reviewers (KW, ZD). The characteristics of the included studies are summarized in Table S2. The following information was extracted: (i) first author name and year of publication, (ii) characteristics of participants (health status, number of participants, age, sex), (iii) study design, (iv) study duration, (v) training frequency, (vi) intervention description, (vii) control group description, (viii) outcomes test method, (ix) main findings.

Physical performance-related indicators in INT and control groups are described as means and standard deviations and were screened and extracted by two reviewers (KW, ZD). A third independent reviewer (RH) was consulted to settle any discrepancies during the data extraction process. If the missing data were still not available, the graph data were extracted using WebPlotDigitizer [21].

### Quality Assessment

Two reviewers (KW and ZD) utilized the revised Cochrane risk-of-bias tool for randomized trials (RoB 2) to assess the risk of bias in each included study [22]. The assessment covered five domains, including the randomization process, deviation from the intended intervention, missing outcome data, measurement of outcome, and selection of reported results. In each domain, the two reviewers evaluated each study as "high risk," "some concerns," or "low risk" based on the signaling questions provided. Any disagreements between the two reviewers (KW, DZ) were resolved through discussion with a third researcher (RH).

### Certainty of Evidence

The certainty of evidence for each outcome was assessed by two authors (KW and DZ) using the Grading of Recommendations Assessment, Development, and Evaluation (GRADE) protocol [23]. The GRADE approach assigns ratings that range from very low to high levels of certainty to determine the quality and strength of the evidence supporting each outcome [24]. To determine the potential downgrading of certainty and strength of recommendations, criteria such as risk of bias, consistency, directness, precision, and publication bias were considered [25]. The GRADE assessment was discussed with the authors to ensure consensus on the interpretation of the findings.

### Synthesis Methods and Statistical Analysis

Statistical analyses were conducted using R (version 4.3.3) [26] with the metafor package 7.0–0 [27]. Meta-analysis was performed when data were available from at least two reports. The physical performance outcomes from each study were reported as mean ± standard deviation (SD). Comparisons were made between the INT group and PT group, and the results of the meta-analysis were based on changes in data (pre- and post-intervention values). In cases where the change values were not provided, the SD of the pre-post change (ΔSD) was calculated assuming a correlation coefficient (R) of 0.5, as previously recommended (ΔSD = √(SD^2^_pre_ + SD^2^_post_-2*R*SD_pre_*SD_post_)) [20]. In cases where a trial was included multiple times in the meta-analysis, the sample size for that trial was divided by the number of times it was used to avoid duplicating its contribution [20]. For data synthesis, random effect models (DerSimonian and Laird) were used. Statistical significance was indicated by a *p*-value less than 0.05. Standardized mean differences (SMDs) and 95% confidence intervals (CIs) were used to analyze static balance, agility, sprinting capacity, jump performance, and strength, considering noncomparable scales and measurements. Dynamic balance and flexibility, on the other hand, were analyzed using weighted mean differences (WMDs) and 95% CIs [28]. Funnel plots visually explained publication bias if at least ten studies were included in the meta-analysis. Egger's linear regression test for funnel plot asymmetry was used to investigate publication bias.

To further investigate the effects of INT versus PT on physical performance in young athletes, we conducted a subgroup analysis. This analysis considered sex, including female, male, and mixed-gender groups; age group, with younger athletes under 14 years and older athletes 15 years and above; duration of the intervention, categorized as less than 12 weeks and 12 weeks or more; and intervention frequency, based on triweekly and biweekly sessions [29]. To enhance the reliability of the findings, a series of sensitivity analyses were conducted to assess the influence of each study, including those with a high risk of bias or outliers that may have impacted the results of the meta-analysis, on the overall conclusions. Outliers are studies with effect sizes that significantly deviate from the norm and can skew results. Sensitivity analyses were performed using a leave-one-out approach. Statistical heterogeneity was evaluated using I^2^ values, which were categorized as low (0 to 25%), moderate (26 to 50%), substantial (51 to 75%), and high (more than 75%) [20].

## Results

### Study Selection

**Fig.1:**
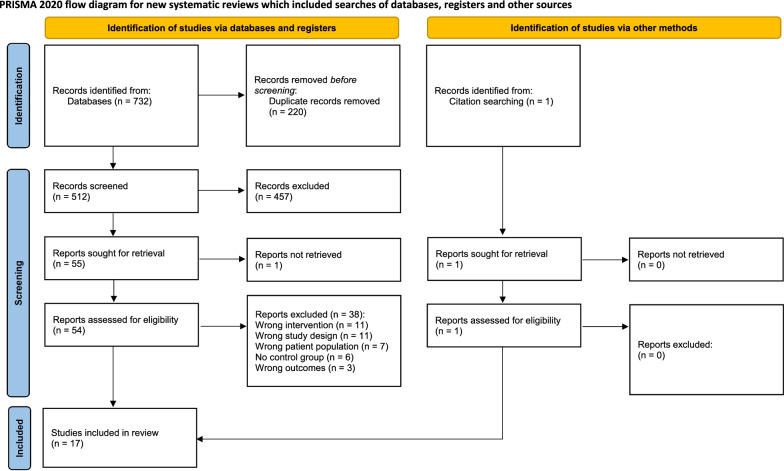
Flowchart of publications included in systematic review and meta-analysis (PRISMA diagram). PRISMA Preferred Reporting Items for Systematic Reviews and Meta-Analyses

Out of 732 records identified for screening in six electronic databases, 220 duplicates were removed, leaving 512 records. A total of 457 records were excluded based on the title and abstract screening as they did not meet the inclusion criteria. Subsequently, 54 full-text articles were reviewed in depth, and 38 records were excluded for the following reasons: (i) studies employed other types of training programs, such as yoga, single plyometric training, and resistance training (n = 11), (ii) studies employed a study design not aligned with our inclusion criteria (n = 11), (iii) subjects, such as adults or non-athletes, did not meet the inclusion criteria (n = 7), (iv) studies did not have a control group (n = 6), (v) studies did not measure the specific outcomes we targeted in our analysis (n = 3). The details are reported in the flow diagram (refer to PRISMA) based on the results of the literature search (Fig. 1).

### Characteristics of Included Studies

Table S2 provides a qualitative description of the included studies. Our analysis included data from 17 RCTs involving a total of 361 participants. The duration of the training interventions ranged from 4 weeks to 10 months (Table S2). The participants in the included studies were young athletes from various sporting backgrounds. Specifically, five studies focused on soccer players [30] [31–34], three included volleyball players [8, 35, 36], one recruited netball players [37], one enrolled cricket players [38], two focused on gymnasts [39, 40], one included basketball players [41], one included rugby players [42], one enrolled tennis players [43], one recruited hockey players [44], and one included ballroom dancers [45]. Regarding the training frequency, eleven studies implemented a training regimen of twice per week [8, 31–33, 35, 36, 38–40, 42, 44], while six studies conducted training sessions three times per week [30, 34, 37, 41, 43, 45].

### Risk of Bias in Studies

The risk of bias for each publication was assessed using the ROB 2 tool. A summary of the overall assessments for all five domains of bias is provided in Table S3. Among the included studies, one study was identified as having a high risk of bias [30], while one study was considered to have a low risk of bias [45]. The remaining fifteen studies were evaluated as having some concerns regarding bias.

It is important to note that all the studies included in the analysis were RCTs; however, only one study provided thorough details on the randomization process. Similarly, only one study explicitly mentioned that participants remained blinded until they arrived at the laboratory to complete the trials. One study was identified as having a high risk of bias, primarily due to concerns related to baseline differences between the intervention groups at the start of the first period. The studies were rated as having some concerns due to a lack of detailed information on the randomization process, and deviations from the intended intervention may have occurred due to contextual factors within the trials.

### Certainty of Evidence

The overall certainty of evidence was assessed using the GRADE tool, and the results are presented in Table S4. The GRADE assessment indicated very low certainty for agility and low certainty for sprinting capacity, indicating that there is limited confidence in the effect estimate. The certainty of evidence for static balance, flexibility, and maximal strength was graded as moderate. This suggests a moderate level of confidence in the effect estimates for these outcomes, although further research could still have an impact on the estimate. Moreover, the certainty of evidence for dynamic balance and jumping performance was graded as high. This indicates a high level of confidence in the effect estimates for these outcomes and suggests the available evidence is robust.

### Results of Data Synthesis

**Fig.2:**
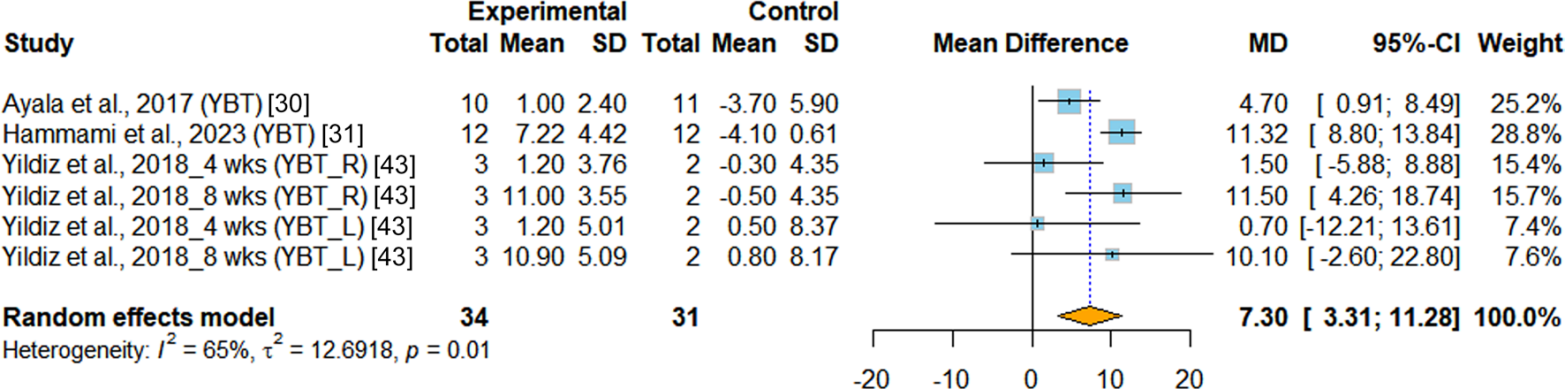
Meta-analysis of the effects of integrative neuromuscular training versus traditional physical fitness training on dynamic balance. MD (mean difference) represents the average difference in the change values between the INT and PT groups. YBT, Y-Balance test. YBT_L, left leg Y-Balance test. YBT_R, right leg Y-Balance test

*Dynamic balance* In the evaluation of dynamic balance among young athletes, three RCTs [30, 31, 43] utilized the Y-balance test as the assessment tool. When comparing the effects of INT and PT on dynamic balance, the results demonstrated a significant improvement favoring INT (MD = 7.29%, 95%CI 3.31 to 11.28, *p* < 0.001, I^2^ = 64.7%). The effect size suggested a notable improvement of approximately 7.29% in the Y-Balance test results, as shown in Fig. 2.

**Fig.3:**
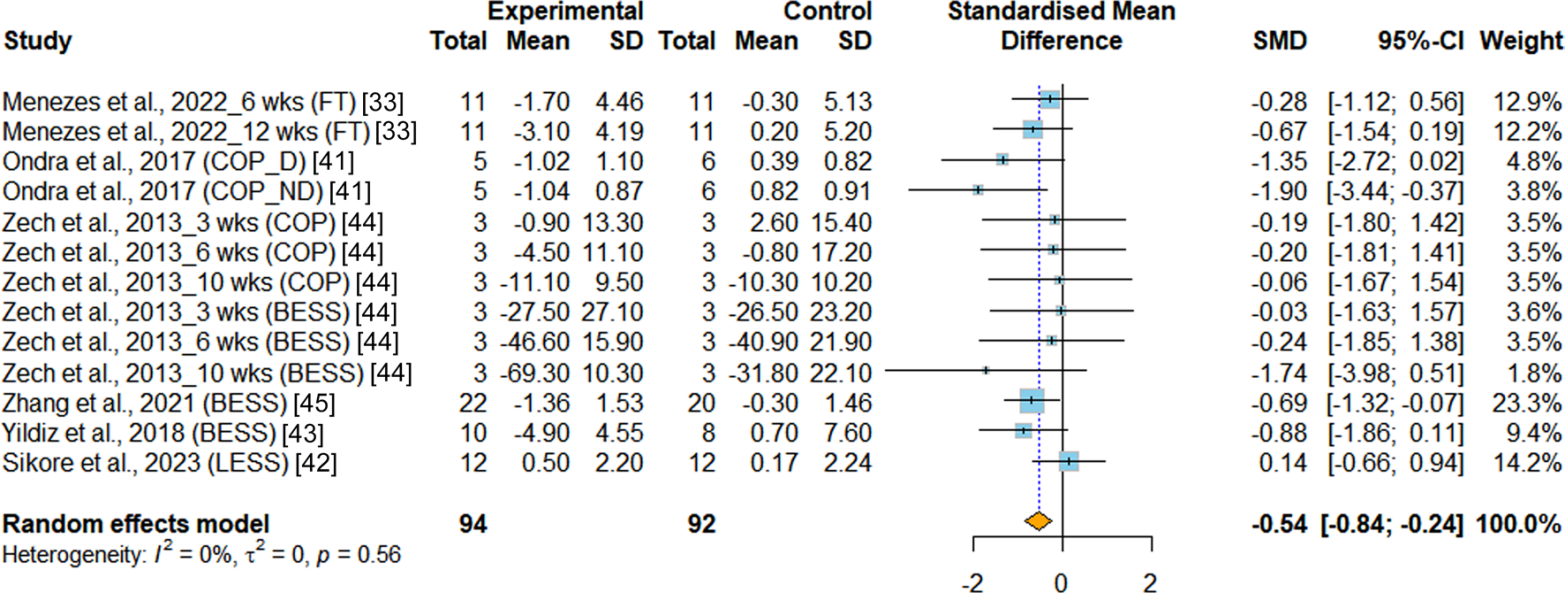
Meta-analysis of the effects of integrative neuromuscular training versus traditional physical fitness training on static balance. SMD (standard mean difference) indicates the standard mean difference in the change values of the INT versus the PT groups. BESS, Balance Error Scoring System. COP, center of pressure. COP_D, center of pressure of dominant limb. COP_ND, center of pressure of non-dominant limb. FT, flamingo test. LESS, Landing Error Scoring System

*Static balance* The meta-analysis included a total of six RCTs [33, 41–45] that assessed static balance (SB) in young athletes. Different measurements were used across the studies: one study utilized the flamingo test [33] with the number of falls as the outcome measure, one study measured postural sway during a single stance using sway velocity [41], three studies employed the Balance Error Scoring System (BESS) [43–45], and one study used the Landing Error Scoring System (LESS) with the test score as the outcome measure [42]. The analysis results indicate a statistically significant improvement in SB when INT is incorporated into young athletes' training compared to PT (SMD = −0.54, 95%CI −0.84 to −0.24, *p* < 0.001, I^2^ = 0.0%) (Fig. 3).

**Fig.4:**
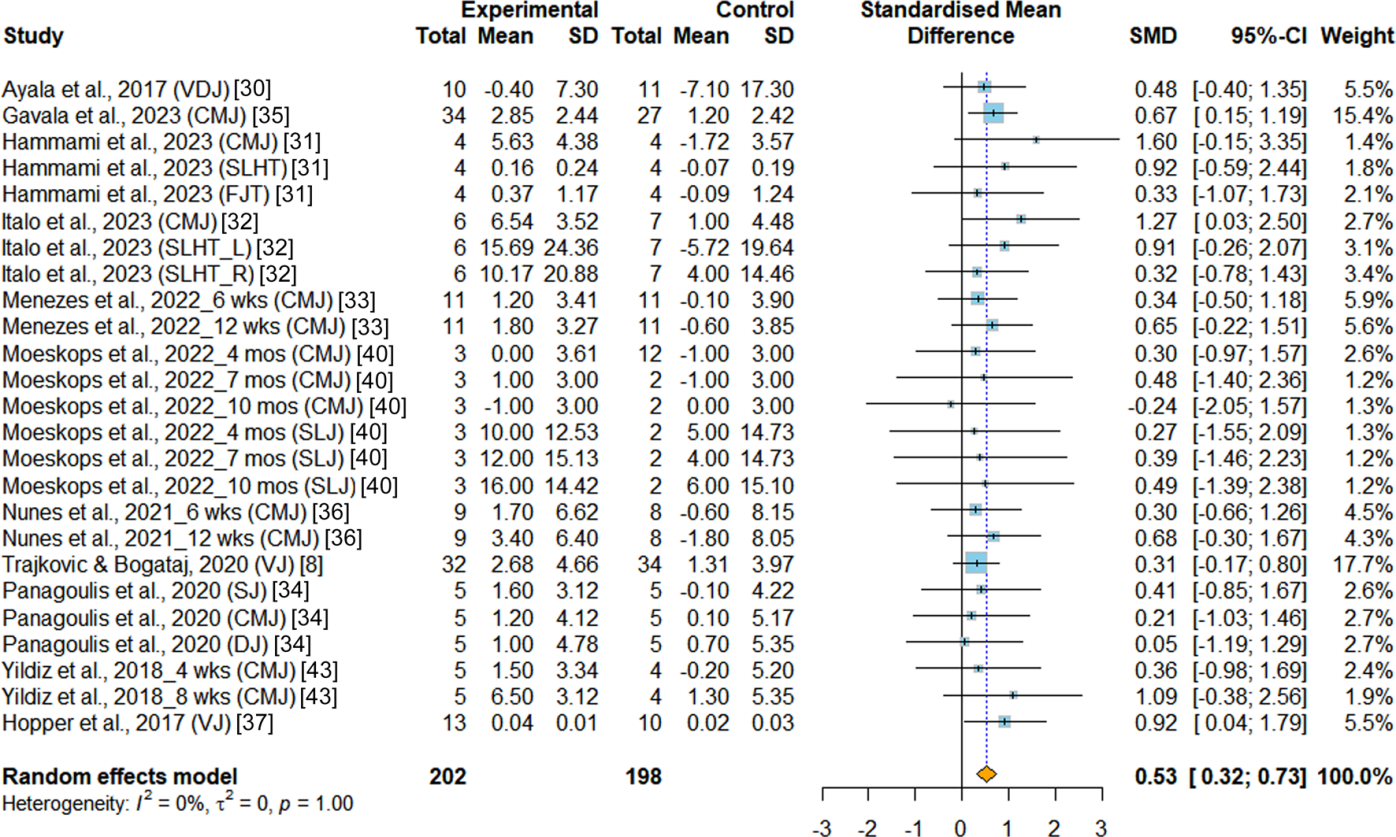
Meta-analysis of the effects of integrative neuromuscular training versus traditional physical fitness training on jumping performance. SMD (standard mean difference) indicates the standard mean difference in the change values of the INT versus the PT groups. CMJ, countermovement jump. DJ, drop jump. FJT, five jump test. SJ, squat jump. SLJ, standing long jump. SLHT, single-leg hop test. SLHT_L, single-leg hop test (left). SLHT_R, single-leg hop test (right). VDJ, vertical drop jump. VJ, vertical jump

*Jumping performance* Jumping performance, a key indicator of power performance, was extensively assessed in the included studies, with ten RCTs [8, 30–37, 40, 43] included in the meta-analysis. Various jump tests were employed to evaluate jumping performance, including the countermovement jump test [31–35, 40, 43], vertical jump test [8], vertical drop jump test [30, 34], single leg hop test [31, 32], five jump test [31], standing long jump [40], and squat jump [34]. According to the result, the INT was associated with significant improvement in jump performance compared with PT (SMD = 0.53, 95%CI 0.32 to 0.73, *p* < 0.001, I^2^ = 0.0%), as shown in Fig. 4. Moreover, the funnel plot showed no indication of publication bias in jumping performance (*p* = 0.58) (Fig.S1).

**Fig.5:**
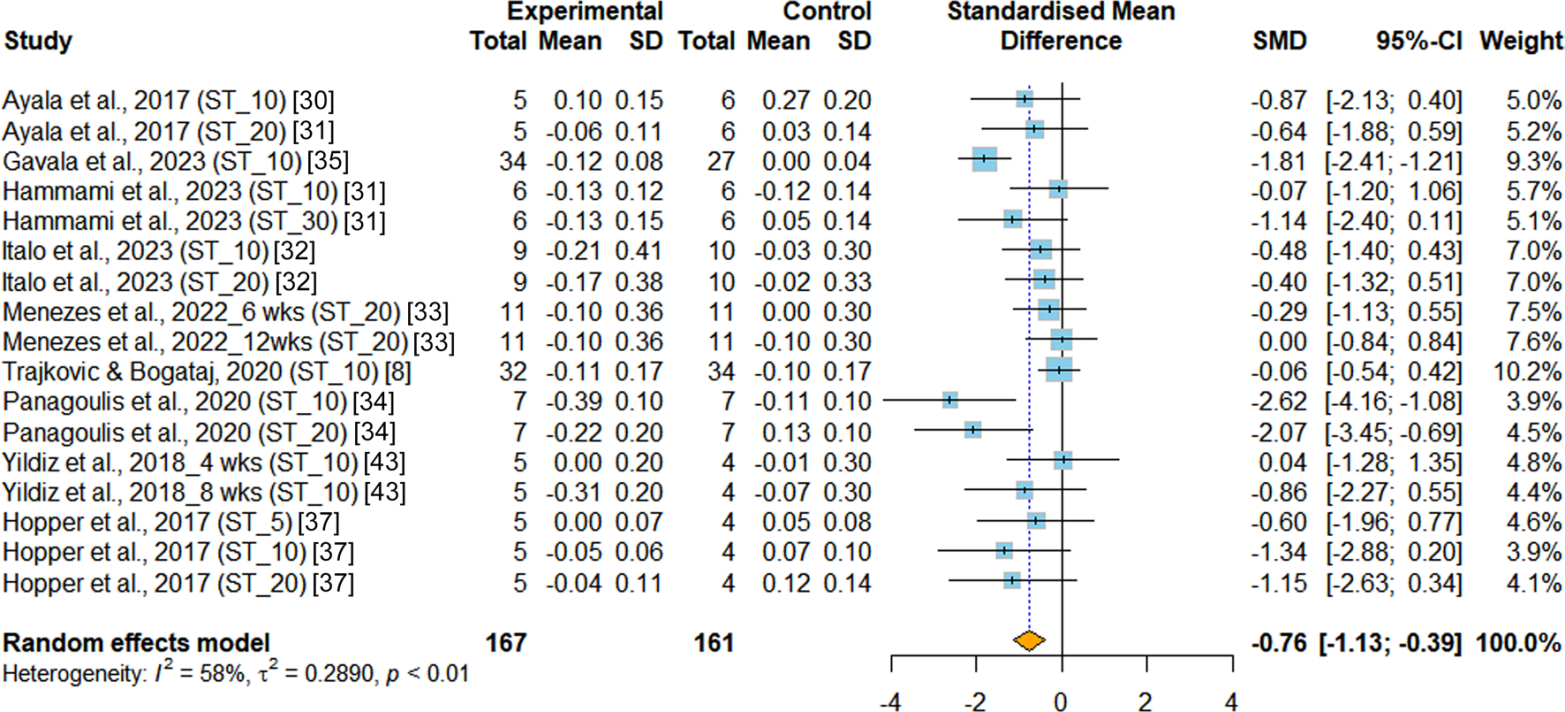
Meta-analysis of the effects of integrative neuromuscular training versus traditional physical fitness training on sprinting capacity. SMD (standard mean difference) indicates the standard mean difference in the change values of the INT versus the PT groups. ST_5, 5-m sprint test. ST_10, 10-m sprint test. ST_20, 20-m sprint test. ST_30, 30-m sprint test

*Sprinting capacity* Data related to sprinting capacity were available in nine RCTs [8, 30–35, 37, 43]. All these studies assessed sprinting capacity based on sprinting time. However, the sprinting distances used varied across the studies, including 5-m [37], 10-m [8, 30, 37], 20-m [30, 33, 34, 37], and 30-m sprints [31]. The meta-analysis results indicated that INT had a significant positive effect on improving sprinting capacity when compared to PT (SMD = −0.76, 95%CI −1.13 to −0.39, *p* < 0.001, I^2^ = 57.6%), as shown in Fig. 5. Additionally, separate analyses for specific distances were conducted. Both the 10-m (MD = −0.12s, 95%CI −0.19 to −0.04, *p* = 0.001, I^2^ = 58.6%) and 20-m sprint (MD = −0.16s, 95%CI −0.27 to −0.05, *p* = 0.004, I^2^ = 32.1%) results indicated that INT had a significant positive effect on improving sprinting capacity (Fig. S2 & S3).

**Fig.6:**
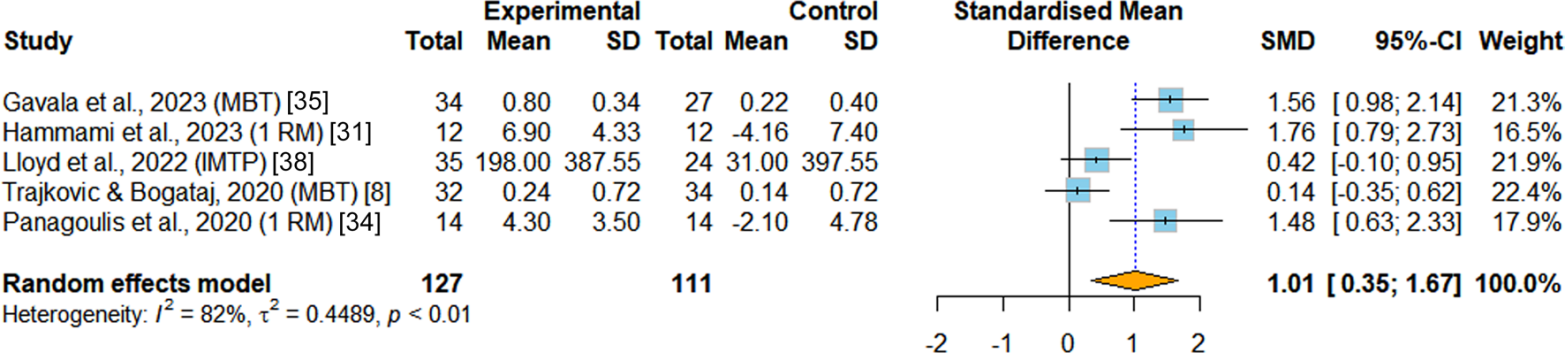
Meta-analysis of the effects of integrative neuromuscular training versus traditional physical fitness training on maximal strength. SMD (standard mean difference) indicates the standard mean difference in the change values of the INT versus the PT groups. 1-RM, one repetition maximum. IMTP, isometric mid-thigh pull. MBT, Medicine ball throw

*Maximal strength* Maximal strength was evaluated in five RCTs [8, 31, 34, 35, 38]. Among these studies, two assessed maximal strength using the one repetition maximum back squat test [31, 34], two utilized the medicine ball throw test [8, 35], and one employed the isometric mid-thigh pull with absolute peak force as the measurement [38]. These various tests were employed to assess and measure maximal strength in the included studies. Overall, pooled data from five RCTs showed significant enhancement favoring INT compared with the PT group (SMD = 1.01, 95%CI 0.35 to 1.67, *p* = 0.002, I^2^ = 81.9%) (Fig. 6).

**Fig.7:**
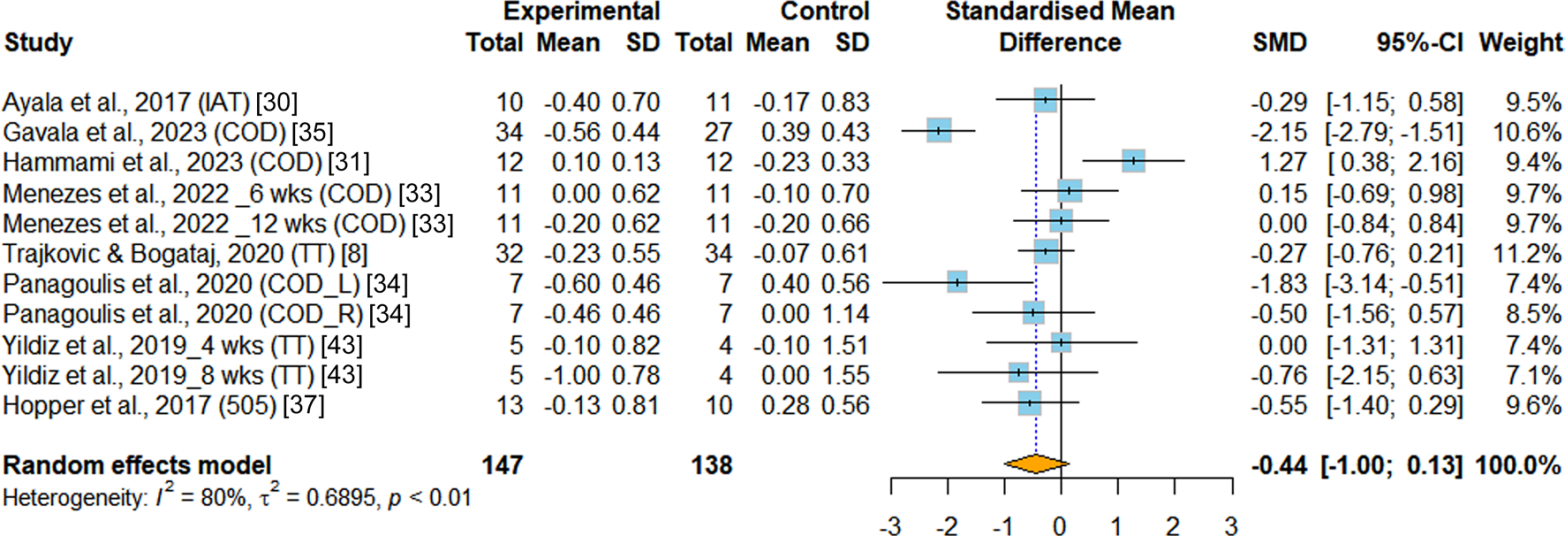
Meta-analysis of the effects of integrative neuromuscular training versus traditional physical fitness training on agility. SMD (standard mean difference) indicates the standard mean difference in the change values of the INT versus the PT groups. COD, change of direction. COD_L, change of direction (left arrowhead). COD_R, change of direction (right arrowhead). TT, t-test. IAT, Illinois agility test. 505, 505 agility test

*Agility* The analysis included a total of eight RCTs that assessed agility [8, 30, 31, 33–35, 37, 43]. All of these studies incorporated change-of-direction tests as part of their assessments, utilizing different modalities to measure change-of-direction performance. However, the analysis did not reveal any significant differences favoring either the INT group or the PT group in terms of improving agility (SMD = −0.44, 95%CI −1.00 to 0.13, *p* = 0.133, I^2^ = 80.0%) (Fig. 7).

**Fig.8:**
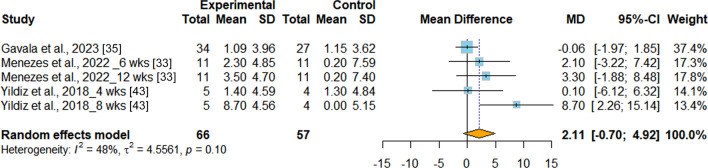
Meta-analysis of the effects of integrative neuromuscular training versus traditional physical fitness training on flexibility. MD (mean difference) represents the average difference in the change values between the INT and PT groups

*Flexibility* Flexibility was evaluated in three RCTs [33, 35, 43], with sit and reach as the test method. According to the result of the meta-analysis, INT did not significantly improve flexibility when compared with PT (MD = 2.11 cm, 95%CI −0.70 to 4.92, *p* = 0.141, I^2^ = 47.9%) (Fig. 8).

### Sensitivity Analyses

A series of sensitivity analyses were conducted to assess the impact of studies with a high risk of bias and outliers on the results of the meta-analysis. The results of these sensitivity analyses are presented in Table S5. For the meta-analysis of agility, the study by Hammami et al. [31] was identified as an outlier that may have influenced the results. After removing this study, there was a decrease in heterogeneity from 80.0 to 73.6%, and the *p*-value changed from 0.133 to less than 0.001. These changes indicate a significant improvement favoring INT over PT in terms of agility. Therefore, the study by Hammami et al. contributed to the higher pooled effect size for agility. Regarding dynamic balance, the study [30] with a high risk of bias was excluded from the analysis. After its removal, the heterogeneity decreased from 64.7 to 51.8%. The improvement of INT for dynamic balance remained significant; however, substantial heterogeneity was still present in the analysis. For maximal strength, removing the study [8] considered an outlier led to a decrease in heterogeneity from 81.9 to 73.6%. This change did not affect the effectiveness of INT on maximal strength, which remained significant. Similarly, in the case of sprinting capacity, removing the study [34] considered an outlier led to a decrease in heterogeneity from substantial to moderate (from 57.6 to 48.4%). However, the effectiveness of INT on sprinting capacity was not affected by this change.

### Subgroup Analyses

The results of the subgroup analysis are presented in Supplementary Material Tables S6–S12. These tables include effect sizes (standardized mean differences and mean differences) with corresponding 95% confidence intervals for each subgroup. Significant differences were observed in dynamic balance for the intervention frequency subgroup (*p* = 0.01) and in sprinting capacity for the gender subgroup (*p* = 0.002).

## Discussion

This is the first systematic review with meta-analysis conducted to directly compare the effects of INT versus PT on physical performance-related outcomes in young athletes. The primary objective of this meta-analysis was to examine whether INT could be a more effective and efficient training strategy compared to traditional methods for young athletes to improve their physical performance-related outcomes. This phase is recognized as a critical developmental stage for athletes, wherein their athletic abilities undergo significant growth and refinement [6, 46]. Our analysis indicates that the inclusion of INT in the daily training regimen of young athletes can lead to substantial improvements in various aspects of their physical performance, including balance, jumping performance, sprinting capacity, and maximal strength. Although initial results for agility and flexibility were not statistically significant, sensitivity analyses revealed that removing an outlier study decreased heterogeneity and resulted in significant improvements in agility favoring INT.

### Effect of INT versus PT on Balance

Our analysis demonstrated a significant improvement in balance with INT compared to PT, as evidenced by significant enhancements in both dynamic balance (*p* < 0.001) and SB (*p* < 0.001), supported by moderate and high certainty of evidence, respectively. The improvement in balance observed in young athletes undergoing INT can be attributed to the training program's "holistic" philosophy. By focusing on enhancing lower limb strength, joint mobility, and proprioceptive control of the body, INT can effectively improve young athletes' balance capabilities [47, 48]. Furthermore, INT involves a variety of exercises that challenge the neuromuscular system in multiple planes of motion, enhancing the integration of sensory input and motor output [49]. This comprehensive approach helps in better recruitment and synchronization of motor units, leading to improved postural control and balance [50, 51].

It is important to note that balance is not fully developed during the youth period and is closely linked to the nonlinear processes of growth and maturation [52–54]. Specifically, individuals undergo substantial physiological changes during the youth phase, including growth spurts and neuromuscular development [55, 56]. These processes can impact balance control and stability, making this a critical area of focus for training interventions. Unlike traditional training methods that prioritize strength and sports skill development, INT focuses on multidirectional movement, neuromuscular control, and proprioceptive training [57, 58]. This approach effectively improves balance in young athletes by exposing athletes to varied movements, enhancing coordination, and refining proprioception [13]. Furthermore, the results of subgroup analyses indicated that longer duration and higher training frequency of INT intervention may lead to more substantial improvements in balance, although the subgroup analysis was not statistically significant. Therefore, it is crucial to determine the appropriate training volume and frequency to optimize the positive effects of INT on balance, ensuring the training is sufficient to maximize performance improvement without overloading young athletes' capacities.

### Effect of INT versus PT on Lower-Body Explosive Power

According to the results of our meta-analysis, we found that INT was associated with significant enhancement of both jumping performance (*p* < 0.001) and sprinting capacity (*p* < 0.001) when compared with the PT group, with high and low certainty of evidence, respectively. Most studies included in the analysis demonstrated positive effects of INT on jumping performance, with one study reporting negative effects [30], which was accounted for in a sensitivity analysis that confirmed the overall result was unaffected. The results for sprinting capacity are consistent with the existing literature, reinforcing the effectiveness of INT in enhancing sprinting ability and lower-body explosive power in young athletes. The superior performance in lower-body explosive power can be attributed to the varied and dynamic nature of INT exercises that promote rapid force production and higher motor unit recruitment [59, 60]. This type of training stimulates the neuromuscular system to adapt more effectively, resulting in improved muscle power and coordination [61].

Furthermore, Nunes et al. observed a significant increase in reverse vertical jump height among junior volleyball players after 20 weeks of INT, and this improvement was maintained even after an 8-week break in training [36]. This suggests that the training-induced adaptations persisted during the detraining period, highlighting the potential for long-term benefits of INT on lower-body explosive power. However, it is important to note that the study highlights the limited research conducted on the effects of INT after detraining and the maximal period of detraining. Understanding the effects of detraining and the optimal duration of training cycles is crucial for designing effective training programs for young athletes. Further research is warranted to investigate the effects of INT after detraining and explore the maximal period of detraining that allows for the maintenance of training-induced adaptations.

### Effect of INT versus PT on Strength

Maximal strength is a crucial component in many athletic activities and can have a significant impact on overall physical performance [62, 63]. In the context of our meta-analysis, the results indicated that INT led to a significant enhancement in maximal performance compared to traditional training methods (*p* = 0.002), with moderate certainty of evidence. This may be because INT considers the balance between anabolic testosterone and catabolic cortisol during strength training, which has been demonstrated to have an impact on maximal strength and hormone ratios [64]. Moreover, INT is widely regarded as a superior training method compared to PT [12, 13]. It specifically aids in developing muscle sensibility, enhancing neuromuscular control, and improving overall body control. These factors are crucial in preventing sports injuries and enhancing young athletes' motor skills. The effectiveness of INT in enhancing strength is due to its capacity to increase motor unit recruitment and synchronization, resulting in greater force production and muscle growth over time [65]. Furthermore, the diverse motor experiences and varied stimuli offered by INT facilitate neural adaptations that improve strength [13].

Hence, in the daily training of young athletes, both traditional training methods (single strength training) and INT can produce good training results for strength qualities if they are performed with proper form and technique [66, 67], and it is advisable for young athletes to integrate INT into their training programs. This recommendation is based on the reduced risk of sports injuries associated with enhanced neuromuscular control that INT offers [68], provided that the training adheres to established norms and movement standards.

### Effect of INT versus PT on Agility and Flexibility

According to the results of the meta-analysis, initially, INT did not show significant improvements in agility (*p* = 0.133) and flexibility (*p* = 0.141) when compared to PT. However, a sensitivity analysis was conducted, which removed a study [31] considered an outlier and inconsistent with most studies. The sensitivity analysis, which reduced heterogeneity from 80.0% to 73.6% and significantly changed the *p*-value from 0.133 to less than 0.001, indicates that after removing the outlier study, INT exhibited a significant improvement in agility compared to traditional training methods. The results showed the importance of sensitivity analysis and strengthened the evidence supporting the effectiveness of INT in enhancing physical performance in young athletes. The enhanced agility observed in INT may be attributed to its impact on proprioception [13, 14]. Proprioception involves the coordination of muscles and joints for maintaining balance and coordinating body movements [69, 70]. It is closely associated with improvements in lower limb muscle strength and trunk stability, which are factors contributing to enhanced agility [58, 71]. Through proprioceptive training, individuals develop heightened body awareness, improved joint alignment, and better control over movement [72]. Specifically, exercises that target the muscles surrounding the joints, particularly in the lower limbs and core, improve stability, allowing for a greater range of motion without compromising joint integrity. This improved stability has a positive influence on agility.

### Methodological Considerations During INT Implementation

As an innovative conceptual training program, there should be more consideration and supervision during the INT implementation to ensure its effectiveness. Firstly, it is crucial to emphasize the importance of a personalized training program that considers factors such as maturation level, training age, technical competency, neuromuscular deficits, sports activities, sex, genetics, and motivation [13]. Additionally, prior to commencing formal training, it is also important to ensure that participants have mastered the correct fundamental technical movements associated with various training exercises to prevent injuries resulting from improper movement patterns. Moreover, during long-term training, athletes' physical performance should be regularly assessed to enable adjustments to the training program based on the assessment results. This approach ensures the comprehensive and effective development of athletes' athletic abilities within the training program. Lastly, the training plan should be tailored to the young athlete's competition schedule while also ensuring training continuity to prevent the loss of training results due to extended periods of detraining.

### Strengths and Limitations

The current systematic review employed a rigorous search strategy, analyzing various physical performance indicators. Unlike previous studies, our review utilized quantitative data analysis methods and focused specifically on the effects of INT, a novel training program not extensively discussed previously. However, this review has several limitations. Despite conducting subgroup analyses based on gender, age group, training frequency, and duration of the intervention, significant heterogeneity remains in study design and assessment tests. This variability can impact the pooled results and necessitates cautious interpretation of our findings. Additionally, the limited number of included studies posed challenges for conducting some subgroup and further analyses to decrease heterogeneity. The included studies often lacked specific details on training dose and volume, hindering our understanding of optimal training parameters. Future research should address these limitations by considering sex-specific analyses, age-specific investigations, and providing detailed training protocols. More studies are needed to standardize methodologies and enhance insights on performance outcomes.

## Conclusion

This study indicates that INT may effectively enhance physical performance in young athletes. These findings could inform coaches, trainers, and practitioners in refining training programs to support the development and performance of young athletes, encouraging a holistic approach to athletic training. Future research could further explore these trends to solidify understanding and application.

## Supplementary Information

Additional file1 (PDF 313 KB)

## Data Availability

The datasets used and/or analyzed during the current study are available from the corresponding author upon reasonable request.

## Abbreviations

BESS: Balance Error Scoring System
Cis: Confidence Intervals
GRADE: Grading of Recommendations Assessment, Development, and Evaluation
IMTP: Isometric Mid-thigh Pull
INT: Integrative Neuromuscular Training
LESS: Landing Error Scoring System
MDs: Mean Differences
PROSPERO: International Prospective Register of Systematic Reviews
PRISMA: Preferred Reporting Items for Systematic Reviews and Meta-Analyses
PT: Traditional Physical Fitness Training
RCTs: Randomized Controlled Trials
RoB 2: Revised Cochrane Risk-of-bias Tool for Randomized Trials
SD: Standard Deviation
SMDs: Standardized Mean Differences
