# Supplementary material for: Comparing the Effects of Integrative Neuromuscular Training and Traditional Physical Fitness Training on Physical Performance Outcomes in Young Athletes: A Systematic Review and Meta-Analysis
Source: Sports Med Open. 2025 Feb 8;11:15. doi: 10.1186/s40798-025-00811-2 (PMC11807040; doi:10.1186/s40798-025-00811-2)
Supplement: Supplementary file 1 — Additional file1 (PDF 313 KB) [file 40798_2025_811_MOESM1_ESM.pdf]

Supplementary Information

Effect of Integrative Neuromuscular Training on Physical Performance in Young Athletes: A Systematic Review and Meta-Analysis

Author Block: Ke-wen Wan[1,2], Zi-han Dai[3], Po-san Wong[3], Robin Sze-tak Ho[3], Bjorn Tsz-king TAM[1,2].

[1] Academy of Wellness and Human Development, Faculty of Arts and Social Sciences, Hong Kong Baptist University, Kowloon Tong, Hong Kong, China
[2]Dr. Stephen Hui Research Centre for Physical Recreation and Wellness, Hong Kong Baptist University, Kowloon Tong, Hong Kong, China
[3]Department of Sports Science and Physical Education, The Chinese University of Hong Kong, Hong Kong, China

Corresponding author: Dr. Bjorn Tsz-king TAM
Academy of Wellness and Human Development, Faculty of Arts and Social Sciences, Hong Kong Baptist University, Kowloon Tong, Hong Kong, China
Email: bjorntam@hkbu.edu.hk
Fax number: +852 3411 5757

Table S1 Searching strategy

**Database name**	**Pubmed**
Full search strategies	(("integrative neuromuscular training" OR "neuromuscular training" OR "comprehensive neuromuscular training" OR "neuromuscular control" OR "neuromuscular program" OR "neuromuscular exercise") AND ("physical performance" OR "athletic performance" OR "motor performance" OR "physical fitness" OR "strength" OR "power" OR "speed" OR "agility" OR "balance" OR "coordination" OR "flexibility" OR "endurance")) AND ("young athletes" OR "adolescent athletes" OR "teen athletes" OR "junior athletes" OR "children athletes" OR "children" OR "adolescents" OR "youth")
Limits and restrictions	Full text, Humans, English
Dates of searches	06/05/2024
Hits	165
**Database name**	**Medline**
Full search strategies	(“integrative neuromuscular training” OR “neuromuscular training” OR “comprehensive neuromuscular training” OR “neuromuscular control” OR “neuromuscular program” OR “neuromuscular exercise”) AND (“physical performance” OR “athletic performance” OR “motor performance” OR “physical fitness” OR “strength” OR “power” OR “speed” OR “agility” OR “balance” OR “coordination” OR “flexibility” OR “endurance”) AND (“young athletes” OR “adolescent athletes” OR “teen athletes” OR “junior athletes” OR “children athletes” OR “children” OR “adolescents” OR “youth”)
Limits and restrictions	Linked Full Text; English Language; Human
Dates of searches	06/05/2024
Hits	10
**Database name**	**Embase**
Full search strategies	(("integrative neuromuscular training" or "neuromuscular training" or "comprehensive neuromuscular training" or "neuromuscular control" or "neuromuscular program" or "neuromuscular exercise") and ("physical performance" or "athletic performance" or "motor performance" or "physical fitness" or "strength" or "power" or "speed" or "agility" or "balance" or "coordination" or "flexibility" or "endurance") and ("young athletes" or "adolescent athletes" or "teen athletes" or "junior athletes" or "children athletes" or "children" or "adolescents" or "youth")).mp. [mp=title, abstract, heading word, drug trade name, original title, device manufacturer, drug manufacturer, device trade name, keyword heading word, floating subheading word, candidate term word]
Limits and restrictions	full text and human and english language
Dates of searches	06/05/2024

Hits	64
**Database name**	**SPORTDiscus**
Full search strategies	**(** “integrative neuromuscular training” OR “neuromuscular training” OR “comprehensive neuromuscular training” OR “neuromuscular control” OR “neuromuscular program” OR “neuromuscular exercise”) AND (“physical performance” OR “athletic performance” OR “motor performance” OR “physical fitness” OR “strength” OR “power” OR “speed” OR “agility” OR “balance” OR “coordination” OR “flexibility” OR “endurance”) AND (“young athletes” OR “adolescent athletes” OR “teen athletes” OR “junior athletes” OR “children athletes” OR “children” OR “adolescents” OR “youth”)
Limits and restrictions	Full Text; Language: English
Dates of searches	06/05/2024
Hits	11
**Database name**	**Web of Science**
Full search strategies	((ALL=(“integrative neuromuscular training” OR “neuromuscular training” OR “comprehensive neuromuscular training” OR “neuromuscular control” OR “neuromuscular program” OR “neuromuscular exercise”)) AND ALL=(“physical performance” OR “athletic performance” OR “motor performance” OR “physical fitness” OR “strength” OR “power” OR “speed” OR “agility” OR “balance” OR “coordination” OR “flexibility” OR “endurance”)) AND ALL=(“young athletes” OR “adolescent athletes” OR “teen athletes” OR “junior athletes” OR “children athletes” OR “children” OR “adolescents” OR “youth”) and English (Languages)
Limits and restrictions	Language: English
Dates of searches	06/05/2024
Hits	331
**Database name**	**PsycINFO**
Full search strategies	(("integrative neuromuscular training" or "neuromuscular training" or "comprehensive neuromuscular training" or "neuromuscular control" or "neuromuscular program" or "neuromuscular exercise") and ("physical performance" or "athletic performance" or "motor performance" or "physical fitness" or "strength" or "power" or "speed" or "agility" or "balance" or "coordination" or "flexibility" or "endurance") and ("young athletes" or "adolescent athletes" or "teen athletes" or "junior athletes" or "children athletes" or "children" or "adolescents" or "youth")).mp. [mp=title, abstract, heading word, table of contents, key concepts, original title, tests & measures, mesh word]

Limits and restrictions	full text and human and english language
Dates of searches	06/05/2024
Hits	30

Table S2 Characteristics of the included studies

Author	Participants, N (sex), age	Study Design	Study duration	Training frequency	Intervention (INT) description	Control group description	Physical performance test method	Main findings
Ayala et al., 2017 [30]	Male youth amateur football players,<br>N = 41;<br>16.8 ± 0.7y	RCT	4 weeks	Three times per week	The FIFA 11+consisted of 3 parts:<br>(a) running exercises;<br>(b) covered 6 exercises, all of which comprised 3 levels of difficulty and were aimed at improving strength, balance, muscle control, and core stability;<br>(c) consisted of advanced running exercises.	Normal warm-up routines try to match the duration of the FIFA 11+.	Range of motion;<br>Single-legged hop limb symmetries;<br>Y-Balance test;<br>10/20-m Sprint test;<br>Agility;<br>Vertical drop jump.	FIFA+ v.s. CON:<br>Dynamic postural control ↑<br>Single-legged hop limb symmetry ↑<br>10 and 20 m sprint times ↓<br>Jump height ↑
Gavala et al., 2023 [35]	Young female volleyball players,<br>N = 61;<br>INT: 12.08 ± 0.11y<br>CG: 11.85 ± 0.95y	RCT	12 weeks	Twice per week	The 1.5-2 h INT program included:<br>(a) balance and agility exercises;<br>(b) core strengthening and stabilization exercises;<br>(c) lower limb plyometric exercises;<br>(d) upper limb and body strengthening exercises with medicine ball.	Volleyball practice.	Sit and reach;<br>10-m sprint speed;<br>t-test COD;<br>Countermovement jump;<br>Medicine ball throw;<br>AAHPERD serve test;<br>Forearm pass test;<br>Setting test.	INT v.s. CG:<br>10-m speed ↑<br>COD score ↑<br>CMJ ↑<br>Medicine ball throw ↑<br>Serve accuracy ↑<br>Passing accuracy ↑<br>Setting accuracy ↑

Hammami et al., 2023 [31]	Youth male soccer players, N = 24; INT: 15.7 ± 0.6y CON: 15.4 ± 0.8y	RCT	8 weeks	Twice per week	The 15-min INT protocol included balance on an unstable surface, strength, plyometric, linear sprint, and COD speed exercises. Each INT session included 5 exercises with 60–120 seconds of rest between sets and exercises.	Standard soccer practice over the same duration.	Y-Balance test; 1RM back squat test; Five jump tests; CMJ test; Single-leg hop test; 30-m linear sprint test; COD with ball.	INT v.s. CON: Composite score of the Y-balance test ↑ 1RM back squat ↑ CMJ height ↑ Five jump test (cm) ↑ Single-leg hop test ↑ 30-m linear sprint time ↓ COD with ball ↓
Hopper et al., 2017 [37]	Junior female netball players, N = 23; 12.17 ± 0.94y	RCT	6 weeks	Three times per week	The 60-min INT program: plyometric exercise and strength training.	Continue with their normal netball training and games.	CMJ test; 20-m sprint test; 505 agility test; NMST.	INT v.s. CON: 10/20-m sprint ↓ 505 change of direction ↓ CMJ height ↑ Peak power values ↑ Total NMST score ↑ The anterior reach and posteromedial reach position ↑ The posterolateral direction (left leg) ↑
Italo et al., 2023 [32]	Young male soccer players, N = 37; 14.31 ± 0.64y	RCT	8 weeks	Twice per week	30 minutes of INT (strength, hold, balance, and stability);	A plan that consisted only in soccer-specific drills.	Hop test; Side test; CMJ; 10-m sprint test; 20-m sprint test.	INT v.s. CON: Hop test left limb ↑ Side test right limb ↑ Side test left limb ↑ CMJ ↑ 10-m sprint (sec) ↓ 20-m sprint (sec) ↓

Lloyd et al., 2022 [38]	Young male cricket athletes, N = 95; Once-weekly INT group: 12.2 ± 1.3y Twice-weekly INT group: 12.0 ± 1.3y CON: 11.7 ± 1.6y	RCT	28 weeks	Twice per week & once per week	(a) 60-minute field-based neuromuscular training session;<br>(b) 60-minute gym-based resistance training session.<br>Both (a) and (b): A variety of fundamental and multijoin dynamic movements that targeted lower and upper body strength and power development, in addition to core strengthening.	No description in the article.	Back Squat Assessment;<br>IMTP;<br>SJ and CMJ tests.	INT (twice weekly) v.s. CON:<br>Back squat assessment total score ↓<br>Absolute peak force (IMTP, SJ) ↑<br>Allometrically scaled peak force (IMTP, SJ) ↑<br>PRFD (CMJ) ↑<br>Relative PRFD (CMJ) ↑
Menezes et al., 2022 [33]	Prepubertal male soccer players, N = 20; 8.2 ± 1.2y	RCT	12 weeks	Twice per week	20 minutes of INT, with exercises to increase muscle power, muscular strength, core strength, and balance, and exercises to improve functional movement skills.	60 minutes of soccer training.	Balance (Flamingo Test);<br>Flexibility (Sit-and-Reach Test);<br>Vertical Jump Height Measurement;<br>20-m Sprint;<br>Running with Change of Direction Speed (Square Test).	INT v.s. CON:<br>Balance ↑<br>Flexibility (cm) ↑<br>Vertical Jump Height (cm) ↑

Moeskops et al., 2018 [39]	Female artistic gymnasts, N = 34; INT: 8.2 ± 1.7y CON: 10.0 ± 1.2y	RCT	8 weeks	Twice per week	The program included: trunk muscular endurance, movement competency, dynamic stabilization, plyometrics, and strength training.	35 min of gymnastics-specific conditioning.	The functional movement screen (Movement proficiency test); Sub-maximal hopping test; DJ; Trunk muscular endurance test.	INT v.s. CON: Extensor muscular endurance ↑ Flexor muscular enduranc ↑ Leg stiffness ↑
Moeskops et al., 2022 [40]	Prepubertal female gymnasts, N = 43; INT: 9.4 ± 1.8y CON: 9.9 ± 1.8y	RCT	10 months	Twice per week	(a) The first phase: movement competency and increasing base levels of muscular strength; (b) The second phase: strength development; (c) The third phase is the gymnasts' strength, power, and speed.	This group followed their gymnastics program.	IMTP; CMJ, DJ, and SLJ; 20-m sprint; Vaulting.	INT: 4 months: IMTP absolute force ↑ SLJ distance ↑ 7 months: IMTP relative force ↑ CMJ ↑ DJ kinetics ↑ 10 months: Speed ↑ Vaulting take-off velocity ↑
Nunes et al., 2021 [36]	Youth volleyball players, N = 32 (19 males); INT: 13.1 ± 0.4y CON: 12.8 ± 0.7y	RCT	12 weeks	Twice per week	15 minutes of INT (dynamic exercise and balance/stabilization exercise).	50 minutes of volleyball training.	CVJH measurement	INT v.s. CON: After 12-weeks: CVJH ↑ Detraining: CVJH ↑

Ondra et al., 2017 [41]	Elite male youth basketball players, N = 21; INT: 17.3 ± 1.3y CON: 16.5 ± 1.8y	RCT	20 weeks	Three times per week	(a) The first battery: five isometric exercises and three dynamic exercises; (b) Second battery: the whole body circuit training (the combination of dynamic and static movements on 6 stations).	A conventional warm-up with the other strength and conditioning coach.	Balance testing	INT v.s. CON: The mean COP velocity in the anterior-posterior direction ↓ The total COP velocity ↓
Panagoulis et al., 2020 [34]	Early adolescent soccer player, N = 28; INT: 11.2 ± 0.5y CON: 11.4 ± 0.57y	RCT	8 weeks	Three times per week	The INT protocol: squats, romanian deadlifts, pistol (Bulgarian) squats, single-leg Romanian deadlifts, box step-ups, core exercises, and dynamic stability exercises using body load, rocker boards, Bosu, and stability balls were performed.	The regular soccer training program.	Ball shooting; Jump ability (CMJ, DJ, and SJ); 20-m sprint; COD; 1RM barbell back squat test.	INT v.s. CON: Time to complete 10m ↓ Time to complete 20m ↓ SJ ↑ CMJ ↑ Arrowhead left ↓ Resistance maximum ↑
Sikora et al., 2023 [42]	Amateur female Rugby Union players, N = 24; INT: 20.05 ± 4.43y CON: 20.04 ± 4.88y	RCT	12 weeks	Twice per week	10 min of INT program (balance, strength, and plyometrics).	10 min of rugby-specific passing drills.	20 bilateral submaximal hopping; 5 maximum hop test; Landing error scoring system.	INT v.s. CON: Reactive Strength Index ↑

Trajković et al.., 2020 [8]	Female volleyball players,<br>N = 66;<br>11.05 ± 0.72y	RCT	10 weeks	Twice per week	Plyometric exercises, coordination ladder, strength training, and finishing with the plank.	The regular volleyball training.	Sprint on 10-m;<br>Modified T-test;<br>Plank;<br>Vertical jump;<br>Medicine ball throw tests.	INT v.s. CON:<br>MQKTK ↑<br>KTK walking backward ↑<br>KTK one-leg jumping ↑<br>Vertical jump (cm) ↑<br>Medicine ball throw (m) ↑
Yildiz et al., 2019 [43]	Male young tennis players,<br>N = 28;<br>9.6 ± 0.7y	RCT	8 weeks	Three times per week	The training program focused on tennis-specific exercises that targeted the muscle groups involved in stroke techniques, incorporating movements on three planes and essential actions such as pushing, pulling, rotating, crouching, lifting, and jumping.	The routine tennis training.	The Y balance test;<br>The balance error scoring system test;<br>CMJ;<br>10-m acceleration;<br>Sit and reach;<br>T-test.	INT v.s. CON:<br>FMS score ↓
Zech et al., 2014 [44]	Male youth field hockey athletes,<br>N = 30;<br>14.9 ± 3y	RCT	20 weeks	Twice per week	The neuromuscular training protocol included running, agility, balance, strength, and plyometric components.	Regular field hockey exercise training sessions.	The star excursion balance test;<br>Balance error scoring system;<br>Time to stabilize.	INT v.s. CON:<br>Balance error scoring system error score ↑

Zhang et al., 2021 [45]	Elite collegiate ballroom dancers, N = 42 (21 males); INT: 19.81 ± 1.72y (male) 19.02 ± 1.97y (female) CON: 20.8 ± 1.24 (male) 20.9 ± 1.58 (female)	RCT	10 weeks	Three times per week	A 45-min INT (balance, strength, and plyometrics).	No description in the article.	Modified-balance error scoring system; Y-Balance test.	INT v.s. CON: Y test results: Y-balance test score on the left/right limb (composite/ posterolateral/ posteromedial) ↑ Double leg/Tandem-Floor ↓ Single leg/double leg/Tandem-Foam ↓

Note. RCT, randomized controlled trial. CON, control group. COP, center of pressure. INT, integrative neuromuscular training group. COD, change of direction. CMJ, countermovement jump. 1RM, one maximal repetition. MNST, netball movement screening tool. IMTP, isometric mid-thigh pull. SJ, squat jump. PRFD, peak rate-of-force development. SLJ, standing long jump. CVJH, countermovement vertical jump height. DJ, drop jump. KTK, Körperkoordinationstest für Kinder. MQKTK, Motor Quotient KTK. FMS, functional movement Screen.

Table S3 Risk of bias assessment in the observational studies included in the systematic review

Study	Risk of Bias Domain					
	**Randomization process**	**Deviations from intended interventions**	**Missing outcome data**	**Measurement of outcome**	**Selection of reported results**	**Overall risk of bias**
Ayala et al., 2017	High	Low	Low	Low	Low	High
Gavala et al., 2023	Some concerns	Low	Low	Low	Low	Some concerns
Hammami et al., 2023	Some concerns	Some concerns	Low	Low	Low	Some concerns
Hopper et al., 2017	Some concerns	Some concerns	Low	Low	Low	Some concerns
Italo et al., 2023	Some concerns	Some concerns	Low	Low	Low	Some concerns
Lloyd et al., 2022	Some concerns	Low	Low	Low	Low	Some concerns
Menezes et al., 2022	Some concerns	Some concerns	Low	Low	Low	Some concerns
Moeskops et al., 2018	Some concerns	Low	Low	Low	Low	Some concerns
Moeskops et al., 2022	Some concerns	Some concerns	Low	Low	Low	Some concerns
Nunes et al., 2021	Some concerns	Some concerns	Low	Low	Low	Some concerns
Ondra et al., 2017	Some concerns	Low	Low	Low	Low	Some concerns
Panagoulis et al., 2020	Some concerns	Low	Low	Low	Low	Some concerns
Sikora et al., 2023	Some concerns	Some concerns	Low	Low	Low	Some concerns
Trajković et al.., 2020	Some concerns	Some concerns	Low	Low	Low	Some concerns
Yildiz et al., 2019	Some concerns	Some concerns	Low	Low	Low	Some concerns
Zech et al., 2014	Some concerns	Some concerns	Low	Low	Low	Some concerns
Zhang et al., 2021	Low	Low	Low	Low	Low	Low

Table S4 Recommendation, Assessment, Development and Evaluation tool for the assessment of certainty of evidence.

Outcome	Study design	Number of participants	Grade assessment					
			Risk of bias	Inconsistency	Indirectness	Imprecision	Publication bias	Certainty of evidence
Agility	7 RCTs	270	Serious[a]	Serious[b]	Not serious	Serious[cd]	Undetected	Very low
Dynamic balance	3 RCTs	63	Serious[a]	Serious[b]	Not serious	Serious[d]	Undetected	Very low
Static balance	5 RCTs	179	Not serious	Not serious	Not serious	Serious[d]	Undetected	Moderate
Flexibility	3 RCTs	123	Not serious	Not serious	Not serious	Serious[cd]	Undetected	Moderate
Sprinting capacity	8 RCTs	299	Serious[a]	Serious[b]	Not serious	Serious[d]	Undetected	Very low
Jumping performance	10 RCTs	359	Serious[a]	Not serious	Not serious	Serious[d]	Undetected	Low
Maximal strength	5 RCTs	238	Not serious	Serious[b]	Not serious	Serious[d]	Undetected	Low

a. High risk of bias with allocation concealment.
b. High heterogeneity.
c. The confidence intervals indicated the potential for important harm or benefit.
d. Sample size not more than 400

Table S5 Sensitivity analysis results

Indicators	Dimension of the analysis	K	SMD/MD	95%CI	Z	P	Heterogeneity
Agility	NA	11	-0.44	-1.00; 0.13	-1.50	0.133	80.0%
	Ayala et al., 2017	10	-0.45	-1.08; 0.17	-1.41	0.158	82.0%
	Hammami et al., 2023	10	-0.61	-1.12; -0.10	-2.35	0.019	73.6%
Dynamic balance	NA	6	7.29%	3.31; 11.28	3.59	< 0.001	64.7%
	Ayala et al., 2017	6	8.09%	3.31; 12.87	3.32	< 0.001	51.8%
Maximal strength	NA	5	1.01	0.35; 1.67	2.99	0.003	81.9%
	Trajkovic & Bogataj, 2020	4	1.25	0.60; 1.89	3.80	< 0.001	73.6%
Static balance	NA	17	-0.76	-1.13; -0.39	-4.01	< 0.001	57.6%
	Ayala et al., 2017	15	-0.77	-1.18; -0.35	-3.65	< 0.001	62.8%
	Panagoulis et al., 2022	15	-0.61	-0.96; -0.26	-3.38	< 0.001	48.4%

NOTE. Studies with large deviations were excluded, and meta-analysis results were analyzed. Studies excluded are listed in the dimension of analysis. NA, no studies were excluded. SMD, standardized mean difference. MD, mean difference.

Table S6 Subgroup analysis for agility

Subgroup	Number of Studies	Effect Size	95%CI	P-value	I^2
Sex					
Female	3	-0.99	[-2.16, 0.17]		90.80 %
Male	6	0.13	[-0.41, 0.67]	0.08	43.60 %
Mixed	2	-1.10	[-2.40, 0.19]		57.90 %
Age					
Younger	9	-0.65	**[-1.22, -0.09]**	0.17	76.10 %
Older	2	-0.49	[-1.04, 2.01]		83.5%
Intervention Duration					
< 12 wks	9	-0.24	[-0.73, 0.36]	0.44	57.5%
≥ 12 wks	2	-1.09	[-3.20, 1.02]		93.8%
Intervention Frequency					
Biweekly	5	-0.22	[-1.31, 0.86]	0.56	91.15 %
Triweekly	6	-0.57	**[-1.01, -0.14]**		0.0%

Table S7 Subgroup analysis for dynamic balance

Subgroup	Number of Studies	Effect Size	95%CI	P-value	I^2
Age					
Younger	4	6.18	**[0.14, 12.22]**	0.66	35.8%
Older	2	8.17	**[1.69, 14.65]**		87.7%
Intervention Frequency					
Biweekly	1	11.32	**[8.79, 13.84]**	**0.01**	--
Triweekly	5	5.54	**[1.93, 9.15]**		19.4%

Table S8 Subgroup analysis for static balance

Subgroup	Number of Studies	Effect Size	95%CI	P-value	I^2
Sex					
Female	1	0.14	[-0.66, 0.94]		--
Male	11	-0.63	**[-1.01, -0.25]**	0.20	0.0%
Mixed	1	-0.69	**[-1.32, -0.07]**		--
Age					
Younger	3	-0.58	**[-1.10, -0.07]**	0.86	0.0%
Older	10	-0.52	**[-0.95, -0.09]**		6.8%
Intervention Duration					
< 12 wks	9	-0.52	**[-0.89, -0.14]**	0.59	0.0%
≥ 12 wks	4	-0.78	[-1.63, 0.08]		58.5%
Intervention Frequency					
Biweekly	3	-0.25	[-0.73, 0.23]	0.13	0.0%
Triweekly	10	-0.72	**[-1.11, -0.34]**		0.0%

Table S9 Subgroup analysis for flexibility

Subgroup	Number of Studies	Effect Size	95%CI	P-value	I^2
Sex					
Female	1	-0.06	[-1.97, 1.85]	0.06	--
Male	4	3.36	[-0.32, 6.41]		23.4%
Intervention Duration					
< 12 wks	3	3.49	[-1.32, 8.31]	0.33	48.9%
≥ 12 wks	2	0.72	[-2.06, 3.50]		29.7%
Intervention Frequency					
Biweekly	3	0.73	[-1.33, 2.79]	0.41	0.0%
Triweekly	2	4.36	[-4.07, 12.79]		71.8%

Table S10 Subgroup analysis for jump performance

Subgroup	Number of Studies	Effect Size	95%CI	P-value	I^2
Sex					
Female	3	0.54	**[0.21, 0.87]**	0.77	0.0%
Male	17	0.57	**[0.27, 0.88]**		0.0%
Mixed	5	0.36	[-0.14, 0.86]		0.0%
Age					
Younger	21	0.51	**[0.29, 0.72]**	0.64	0.0%
Older	4	0.66	**[0.04, 1.29]**		0.0%
Intervention Duration					
< 12 wks	16	0.50	**[0.25, 0.75]**	0.77	0.0%
≥ 12 wks	9	0.57	**[0.22, 0.92]**		0.0%
Intervention Frequency					
Biweekly	18	0.52	**[0.29, 0.76]**	0.97	0.0%
Triweekly	7	0.53	**[0.11, 0.95]**		0.0%

Table S11 Subgroup analysis for maximal strength

Subgroup	Number of Studies	Effect Size	95%CI	P-value	I^2
Sex					
Female	2	0.84	[-0.55, 2.23]	0.69	92.6%
Male	2	1.03	[-0.28, 2.33]		82.5%
Mixed	1	1.48	**[0.63, 2.33]**		--
Age					
Younger	4	0.86	**[0.14, 1.58]**	0.14	83.3%

Older	1	1.76	**[0.79, 2.72]**		--
Intervention Duration					
< 12 wks	4	0.87	**[0.10, 1.63]**	0.16	78.0%
≥ 12 wks	1	1.56	**[0.98, 2.14]**		--
Intervention Frequency					
Biweekly	4	0.91	**[0.13, 1.70]**	0.33	84.4%
Triweekly	1	1.48	**[0.63, 2.33]**		--

Table S12 Subgroup analysis for sprinting capacity

**Subgroup**	**Number of Studies**	**Effect Size**	**95%CI**	**P-value**	**I^2**
Sex					
Female	5	-0.95	**[-1.73, -0.17]**	**0.002**	80.5%
Male	10	-0.40	**[-0.73, -0.07]**		0.0%
Mixed	2	-2.31	**[-3.34, -1.29]**		0.0%
Age					
Younger	13	-0.80	**[-1.25, -0.34]**	0.70	66.7%
Older	4	-0.65	**[-1.26, -0.04]**		0.0%
Intervention Duration					
< 12 wks	15	-0.65	**[-0.99, -0.31]**	0.76	31.4%
≥ 12 wks	2	-0.93	[-2.70, 0.84]		91.5%
Intervention Frequency					
Biweekly	8	-0.54	**[-1.03, -0.56]**	0.14	71.3%
Triweekly	9	-1.06	**[-1.57, -0.56]**		18.8%

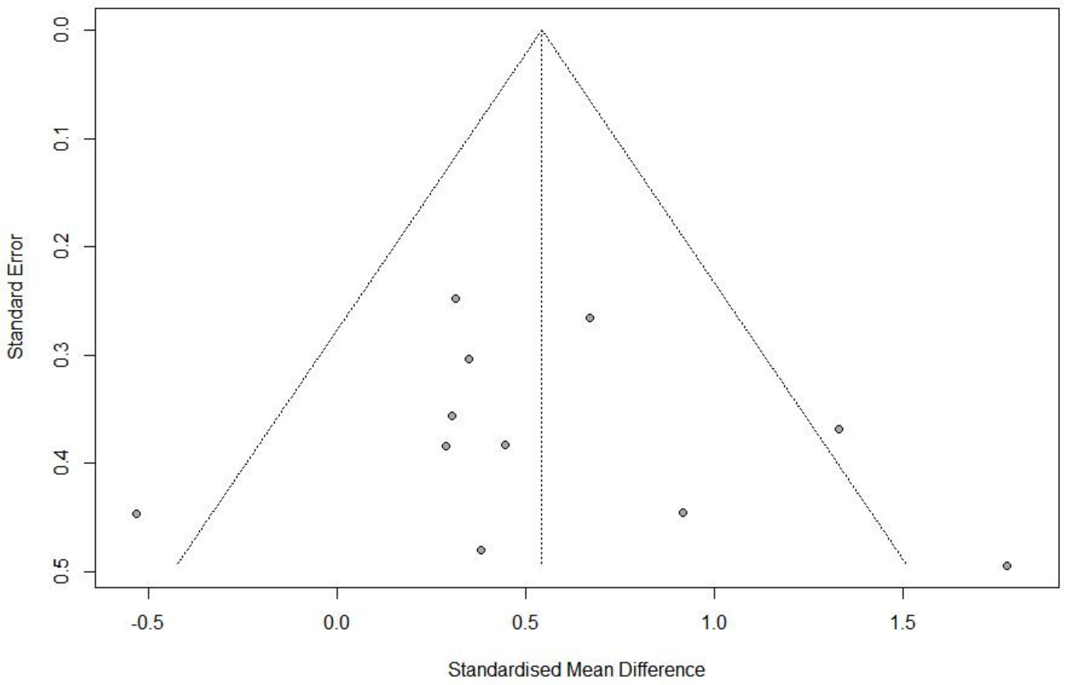

Fig.S1 Funnel plot for publication bias detection on jump performance. The funnel plot shows the observed standardised mean differences (on the x-axis) against standard errors (on the y-axis).

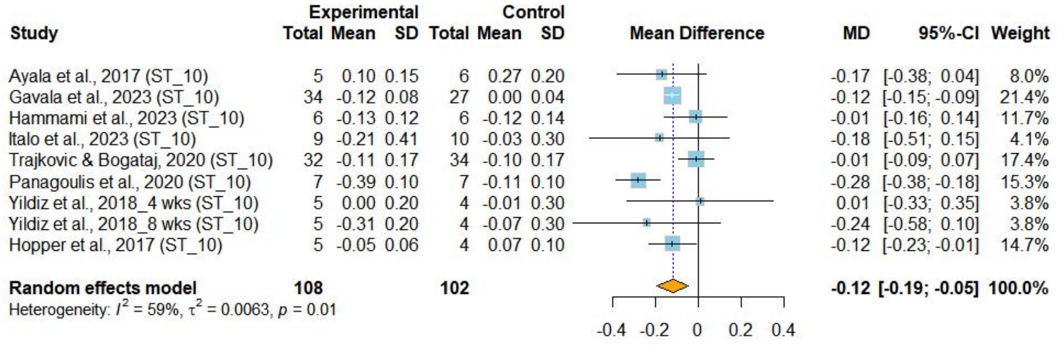

Fig.S2 Meta-analysis of the effects of integrative neuromuscular training versus traditional physical fitness training on 10m sprint. MD (mean difference) represents the average difference in the change values between the INT and PT groups. ST_10, 10-m sprint test.

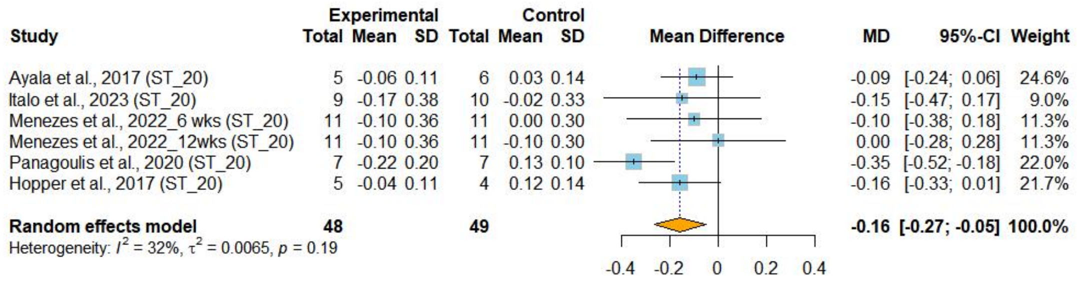

Fig.S3 Meta-analysis of the effects of integrative neuromuscular training versus traditional physical fitness training on 20m sprint. MD (mean difference) represents the average difference in the change values between the INT and PT groups. ST_20, 20-m sprint test.
